# Sequence and Apoptotic Activity of VacA Cytotoxin Cloned from a *Helicobacter pylori* Thai Clinical Isolate

**DOI:** 10.1155/2014/398350

**Published:** 2014-04-02

**Authors:** Muhammad Junaid, Sarbast Al-Gubare, Muhammad Yousef, Mathukorn Na Ubol, Somphob Leetachewa, Chatchai Muanprasat, Chanan Angsuthanasombat, Wanpen Chaicumpa, Niaz Ali, Gerd Katzenmeier

**Affiliations:** ^1^Department of Pharmacy, Division of Pharmacology, University of Malakand, Khyber Pakhtunkhwa 18550, Pakistan; ^2^Bacterial Protein Toxin Research Cluster, Institute of Molecular Biosciences, Mahidol University, Salaya, Nakornpathom 73170, Thailand; ^3^Department of Physiology, Faculty of Science, Mahidol University, Bangkok 10400, Thailand; ^4^Department of Parasitology, Faculty of Medicine, Siriraj Hospital, Mahidol University, Bangkok 10700, Thailand; ^5^Department of Pharmacology, Institute of Basic Medical Sciences, Khyber Medical University, Peshawar 25000, Pakistan

## Abstract

The vacuolating cytotoxin VacA produced by *Helicobacter pylori* induces the formation of large cytoplasmic vacuoles in host gastric epithelial cells as well as a release of cytochrome C from mitochondria resulting in cell apoptosis. Considerable sequence diversity in VacA relating to different degrees of disease severity is observed with clinical samples from a multitude of geographic places. In this study we describe expression in *Escherichia coli*, purification to homogeneity and *in vitro* assay of its apoptotic activity of a VacA toxin from a *H. pylori* isolate of a Thai patient with gastrointestinal lymphoma. Sequencing revealed that the deduced amino acid sequence of the cloned Thai isolate VacA is similar to *H. pylori* s1/m2 type strains. The percent sequence similarity to the model strain 60190 was lower due to the presence of extra amino acids in the mid (m) region. The purified VacA toxin exhibited significant apoptotic activity on both T84 and MDCK epithelial cell lines, as revealed by DAPI staining, whereby the observed activity was significantly higher on MDCK cells. These findings could relate to a modulation of VacA activity on host cells in the Thai isolate-VacA toxin that may differ from those of the model strain.

## 1. Introduction

*Helicobacter pylori *is a Gram-negative spiral-shaped bacterium, which causes serious chronic infectious diseases that damage gastric structure, and function and lead to gastric atrophy, peptic ulcer disease, gastric adenocarcinoma and primary gastric cell lymphoma [1, 2]. About half of the world's population is infected with* H. pylori* making this organism the most prevalent bacterial pathogen which is known to date [3, 4].

The vacuolating cytotoxin A (VacA) is one of the major virulence factors released by* H. pylori*. VacA targets not only epithelial cells, but also cells of the immune system where it induces immunosuppression [5]. The* vacA *gene is present in all strains and encodes a ~140-kDa protoxin [6] which upon proteolytic processing produces the mature ~90 kDa (~821 amino acids) toxin [7]. The mature toxin can undergo proteolytic cleavage into fragments of 33 and 55 kDa which represent 2 domains of VacA [8–10]. The p33 domain is involved in membrane insertion and ion-channel formation, whereas the p55 domain has a role in receptor binding and toxin oligomerization [11, 12]. Two receptor-like protein tyrosine phosphatases, RPTP*α* and RPTP*β*, were identified as cellular receptors involved in VacA uptake [13, 14].

The production of cytotoxin activity can vary considerably among* H. pylori *strains due to extensive sequence polymorphisms in the* vacA *gene [15]. The most variable region corresponds to a ~800-bp sequence located in the middle of the p55 domain (“mid” or “*m*” region). Various* m* sequences have been grouped into two families of alleles,* m*1 and* m*2, whereby strains carrying the* m2* phenotype appear to have limited vacuolating activity [16]. Additional sequence variations are located in the “*s*” region which includes the N-terminal signal sequence of the mature VacA toxin [17]. Two main allelic families are recognized, designated* s*1 and* s*2. All possible combinations of these polymorphic regions (*s*1/*m*1,* s*1/*m*2,* s*2/*m*1, and* s*2/*m*2) have been detected in clinical isolates of* H. pylori*, although* s*2/*m*1 forms occur rarely. Strains containing a type* s2* allele reportedly are unable to induce vacuolation with specific cell types [17].

A third polymorphic site within the* vacA* sequence was recently identified and designated “*i*” (intermediate) region [18]. The “*i*” region is located within the p33 domain and 2 types,* i*1 and* i*2, were commonly found in clinical isolates of* H. pylori*. Vacuolation assays demonstrated that vacuolating activity of* s*1/*m*2 VacA was dependent on the presence of the “*i*” region, thereby suggesting a pivotal role for VacA activity.

Vacuolating cytotoxin purified from* H. pylori *causes mitochondrial damage leading to apoptotic cell death and it was speculated that differences in gastric mucosal cell apoptosis among* H. pylori-*infected persons may be caused by differences in the structure and activity of VacA allelic forms [19].

In the present study, we constructed an expression system to obtain sequence information and to analyze the apoptotic activity of a* H. pylori* VacA toxin isolated from a Thai patient with gastric lymphoma (“*Thai isolate*”). We were seeking to understand how genetic differences and sequence polymorphisms between* m*1 and* m*2 types of* vacA* alleles are related to the Thai isolate VacA and how these could possibly influence the apoptotic activity of this protein.

## 2. Materials and Methods

### 2.1. Cloning and Sequence Analysis of* vacA* Gene from a Thai Patient Isolate

*H. pylori* was cultured from a gastric biopsy sample from a Thai patient with diagnosed gastric lymphoma (62-year-old male) obtained at Vichaiyut Hospital, Bangkok, Thailand, on the surfaces of horse blood agar plates (10% horse blood in Casman agar base (BBL Microbiology Systems, Cockeysville, MD)), which were incubated in an atmosphere of 5% oxygen and 10% carbon dioxide for 72 h at 37°C for up to 5 days [20–22]. Chromosomal DNA was extracted as described previously [23] and used as template for PCR amplification of* vacA*. The following pair of primers derived from the sequence of* H. pylori* model strain 60190 was used:

SJ_*vacA*1F: 5′-CATGCCATGGCCTTTTTTACAACCGTGATCA-3′ (underlined sequence represents the* Nco*I restriction site) and SJ_*vacA*821R: 5′-TGCACTGCAGAGCGTAGCTAGCGAAACGC-3′ (underlined sequence represents the* Pst*I restriction site). Oligonucleotides were purchased from Sigma-Aldrich, Singapore.

PCR was performed with* Pfu* DNA polymerase (Fermentas Life Sciences, USA) using a thermal cycler GeneAmp PCR system Model 2400 (Perkin Elmer Cetus, USA), with predenaturing (98°C, 2 min), 25 cycles of denaturing (98°C, 0.1 min), annealing (65°C, 0.3 min), extension (72°C, 1.3 min), and final extension (72°C, 7 min). The resulting PCR products were subjected to agarose gel electrophoresis, and the 2.5-kb fragment representing the mature full-length* vacA* toxin gene (p88) was excised and purified by QIAquick gel extraction kit (QIAGEN, Germany).

The purified* vacA* PCR product and pTrcHisA (4.4 kb) vector (Invitrogen, USA) were digested with restriction enzymes* Nco*I and* Pst*I and purified by QIAquick purification kit. Insert DNA and pTrcHisA vector were combined at a 10 : 1 molar ratio in a ligation reaction containing 1× ligation buffer (50 mM Tris-HCl, pH 7.6, 10 mM MgCl_2_, 1 mM ATP, 1 mM DTT, 25% (w/v) polyethylene glycol 8000) and five units of T4 DNA ligase (Gibco BRL, USA) in a final volume of 20 µL and incubated overnight at 14°C, resulting in pTrcHisA/VacA carrying a C-terminal (His)_6_ tag and a translation initiation methionine residue at the N-terminus. The correct sequence of the recombinant pTrcHisA/VacA construct was verified by restriction digestion and DNA sequencing analysis (Macrogen Inc., South Korea).

### 2.2. Expression and Purification of the Recombinant VacA Protein

Recombinant plasmid containing the* vacA* insert (pTrcHis2A/VacA) was transformed into* Escherichia coli* TOP10 (GIBCO BRL, USA). To determine optimum conditions for the expression of the VacA protein, the* E. coli* cells were incubated at 37°C in one liter LB medium containing 100 *μ*g mL^−1^ ampicillin. At OD_600_ = 0.5, expression was induced by isopropyl-*β*-D-thiogalactopyranoside (IPTG, 0.1 mM final concentration). Protein biosynthesis was assayed at different incubation times (0 h, 1 h, 4 h, and 6 h and overnight) and temperatures (18, 25, 30, and 37°C). Cells were harvested by centrifugation (6000 ×g, 4°C, 10 min) and the pellet was resuspended in 30 mL lysis buffer (0.1 M Tris-HCl, pH 7.5, 0.3 M NaCl, 0.25 mg mL^−1^ lysozyme, 10 mg mL^−1^ DNase, and 5 mM MgCl_2_). Cells were lysed on ice by sonication using an Ultrasonic Processor XL (Misonix Inc., USA). The cell lysate was subjected to centrifugation (15000 ×g, 4°C, 30 min), insoluble material was pelleted by centrifugation (15000 ×g, 4°C, 20 min), and the soluble fraction was filtered through a 0.22 micron pore-size filter (Pall Corporation, USA).

Histidine-tagged VacA protein was purified by immobilized metal ion affinity chromatography (IMAC), using nickel-sepharose HisTrap HP 5-mL columns (GE Healthcare, Sweden). The columns were preequilibrated with 5–10 column volumes of sample buffer containing 100 mM Tris-HCl, pH 7.5, 300 mM NaCl, and the sample was loaded at a flow rate of 1 mL min^−1^, using a FPLC pump (AKTA FPLC system, GE Healthcare). The column was washed with 10 column volumes of degased washing buffer (100 mM Tris-HCl, pH 7.5, 300 mM NaCl, 10 mM imidazole) and the protein was eluted with elution buffer (100 mM Tris-HCl, pH 7.5, 300 mM NaCl, 100 mM imidazole) at flow rate of 1 mL min^−1^. Elution was monitored by absorbance at 280 nm using a UV detector (AKTA FPLC system, GE Healthcare) and fractions of 2 mL were collected. From each fraction, 20 *μ*L were analysed on SDS-PAGE (10% gel). Western blotting was performed using anti-VacA rabbit antiserum (Invitrogen, USA) at 1 : 2000 dilution with alkaline phosphatase color detection.

Fractions containing VacA were desalted by stepwise dialysis at 4°C by using SPECTRA/POR dialysis membranes (6–8 kDa MWCO) (Spectrum Medical Industries, Inc. MA, USA), against three batches of a 100-fold sample volume buffer A (100 mM Tris-HCl, pH 7.5, 200 mM NaCl), one batch of a 100-fold volume buffer B (100 mM Tris-HCl, pH 7.5, 100 M NaCl), and one batch of a 100-fold volume of buffer C (50 mM Tris-HCl, pH 7.5). Purified VacA was further concentrated to 1.0 mg mL^−1^ by centrifugal filter devices (Centricon 15 mL, 30-kDa MWCO, Millipore, USA) at 4°C. Protein concentrations were determined with a Bradford protein microassay (Bio-Rad, USA) using bovine serum albumin (Sigma, USA) as calibration standard. Samples were used immediately or stored in 50 mM Tris-HCl, pH 7.5, 50% (v/v) glycerol, at −20°C.

### 2.3. Assay of VacA Apoptotic Activity

Human colonic adenocarcinoma (T84) and Madin-Darby canine kidney (MDCK) epithelial cells, originally purchased from the American Type Culture Collection (Manassas, USA), were grown as monolayers in a 1 : 1 mixture of Dulbecco's modified Eagle's Medium/Nutrient Mixture F-12 Ham (DMEM-Ham) (Sigma, USA) supplemented with 50 U mL^−1^ penicillin, 50 *μ*g mL^−1^ streptomycin, and 5% foetal bovine serum. The culture medium was replaced every other day. Monolayers were subcultured by trypsinization with 0.25% (w/v) trypsin and 5.3 mM EDTA in Ca^2+^- and Mg^2+^-free phosphate-buffered saline (PBS) and plated on coverslips at a density of 10^5^ cells mL^−1^ to study apoptosis caused by VacA. T84 and MDCK cells were seeded in 75 mL flasks at 37°C in a humidified atmosphere of 5% CO_2_.

Apoptosis of the colonic epithelium was assessed using a nuclear stain, 4′,6-diamidino-2-phenylindole dihydrochloride (DAPI) (Sigma, USA). Cells were placed on coverslips at a density of 5 × 10^5^ cells per well in DMEM medium and kept at the incubator. At the time of experiment, old medium was removed and cells were incubated with either 150 µg mL^−1^ VacA-containing medium or serum-free medium (as a control) for 24 h. Cells were washed in PBS and fixed with 60 *μ*L of 4% paraformaldehyde for 8 min at 4°C followed by three-time washing with 60 *μ*L of 1× PBS. Cells were incubated with 60 *μ*L of 0.1% Triton X-100 for 10 min and nonspecific binding sites were blocked by adding 2% skimmed milk powder for 1 h. Cells were washed in PBS three times, 10 min each. Finally, cells were stained with 50 *μ*L of DAPI (1 : 1000 dilution in blocking buffer) for 15 min and mounted using 50% glycerol. The signals were visualized at wavelength 350/460 nm (excitation/emission) by using a fluorescence microscope (model IX71, Olympus, Japan). Binding of DAPI to dsDNA produced a ~20-fold fluorescence enhancement and a minimum of 200 cells was counted for each sample and the control by visual inspection of microscopic images. Results are the mean of three independent experiments and data are represented as mean ± standard error of the mean.

## 3. Results and Discussion

### 3.1. Sequence and Allele Type of VacA Toxin Gene from Thai Patient Isolate

The 2.5-kb* vacA* gene sequence encoding the mature VacA toxin was obtained by PCR amplification using* H. pylori* genomic DNA extracted from a patient isolate as template. VacA is the product of a single gene that encodes a 140-kDa precursor protein which, upon proteolytic removal of the N-terminal signal sequence, yields the mature 88-kDa toxin containing alanine as N-terminal amino acid residue [7]. An N-terminal methionine residue was included to ensure proper initiation of translation in the recombinant* E. coli* host and a C-terminal (His)_6_ sequence was added for purification purposes.

**Figure 1:**
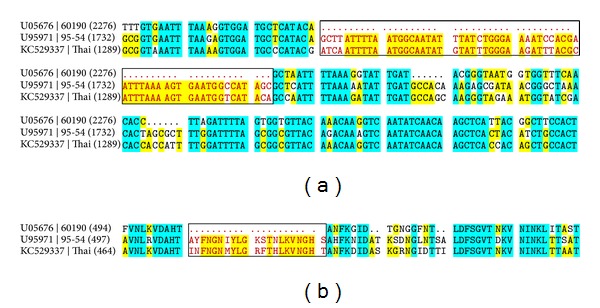
Partial nucleotide sequence and deduced amino acid sequence in the* m* region of recombinant* vacA* toxin gene from Thai isolate (rVacA) aligned with sequences of* H. pylori* model strain 60190 (*s*1/*m*1) and strain 95-54 (*s*1/*m*2). (a) Nucleotide sequences identical in the 3 strains are in blue, and nucleotide sequences identical between 2 strains are shown in yellow. The black box shows the insertion in the middle region of the Thai isolate* vacA* gene. (b) Amino acid sequences identical in the 3 strains are in blue, and amino acid sequences identical between 2 strains are shown in yellow. The black box shows the insertion in the middle region of the Thai isolate* vacA* gene.

The construct was analyzed by nucleotide sequencing and alignment with sequences of* H. pylori* strain 60190 (*s*1/*m*1 allelic type)* vacA* sequence (GenBank accession number U05676), and strain 95-54 (*s*1/*m*2 allelic type) (GenBank accession number U95971) (Figure 1). The sequence of the Thai isolate was deposited in GenBank at accession number KC529337. Sequence analysis revealed that the sequence of the cloned Thai* vacA* gene encompasses 2577 bp encoding a mature VacA toxin of 859 amino acid residues. The sequence of the Thai isolate* vacA* gene is identical to a recently described strain isolated in China (CHN1811a; GenBank accession number AF050326). It shows ~82% identity to the toxigenic* H. pylori s*1/*m*2 strain 95-54 and identity to the* s*1/*m*1 model strain 60190 is only ~53% (Figure 1(a)).* H. pylori* strain 95-54 encodes an unusually large VacA toxin of 1323 amino acid residues and has been shown to possess an* s*1/*m*2 allelic phenotype [17].

Comparison of deduced amino acid sequences showed a well conserved p33 domain among the surveyed strains containing* m*1 and* m*2 alleles and a diversification region which differentiates* m*1 and* m*2 allelic types. Residue D455 is located at the beginning of the mid region in* H. pylori* strain 60190 and it was proposed that this area represents a receptor binding site common to all* m*1 and* m*2 strains [24].

The most notable difference between* H. pylori* strain 60190 and the Thai strain is the sizable insertion of 21 amino acid residues in the middle region making the Thai isolate an* m*2 type strain (Figure 1(b)). The structural consequences of this insertion are not well understood at present. In particular, it is unclear whether the inserted sequence directly participates in receptor binding as both RPTP*α* and RPTP*β* are recognized by the* m*2 type of* vacA* alleles [25].

Numerous studies have demonstrated a correlation between the allelic types of “*m*” and “*s*”regions and the occurrence of gastroduodenal diseases in humans; however, the pathophysiological mechanisms involved in these associations are characterized only to a limited extent [17, 26–35]. A study on the clinical relevance of VacA genotypes corroborated that VacA type* s*2 strains are rarely associated with peptic ulceration [17]. As regions of diversity in* vacA *alleles comprise an ample portion of the gene, structural differences between type* m*1 and type* m*2 gene products could give rise to differences in cytotoxin phenotypes affecting receptor recognition, internalization, and vacuolation. Cell-specific binding has been attributed to differences in the* m*1 and* m*2 alleles. Strains encoding* s*1/*m*1* vacA* genes typically produce VacA with cytotoxic activity on human cervical carcinoma HeLa cells, whereas* m*2-type VacA could induce vacuoles in primary cultured human gastric cell lines as well as nongastric epithelial RK13 cells, but not in HeLa cells [36].

It is interesting to note that the third polymorphic determinant within the Thai isolate* vacA* sequence, the “*i*” region, shows marked differences to strain 95-54, whereas it shares greater homology to the other model strains. How the “*i*” region functions in the context of VacA allelic polymorphisms needs to be elucidated in future studies.

Currently, most* in vitro* studies utilize the s1/m1 type of VacA. Therefore, analysis of the biological effects of the s1/m2 type VacA as described herein could contribute to a better understanding of the relation between toxin activity and their allelic variations. We are currently working on a comparative analysis of toxin activity and structural differences from the s1/m1 model strain 60190 and the s1/m2 Thai isolate.

### 3.2. Expression and Purification of the Recombinant VacA Protein

The recombinant plasmid pTrcHis2A/VacA directed expression of the VacA toxin in* E. coli* TOP10 upon induction with IPTG. Optimization of expression conditions was attempted to achieve increased levels of expression. Time-dependent expression of VacA was analyzed by SDS-PAGE of crude lysate and subsequent Western blotting with commercially available anti-VacA antiserum. It is interesting to note that growth of the recombinant stain carrying pTrHis2A/VacA ceased after addition of IPTG, and over a period of 14 h, no further increase in bacterial growth rate was observed, thereby suggesting cytotoxic effects of the recombinant VacA toxin on* E. coli* cell growth (data not shown). At lower temperatures (18−25°C), reduced amounts of recombinant VacA were detected after incubation for 14 h and, consequently, expression was performed at 37°C.

**Figure 2:**
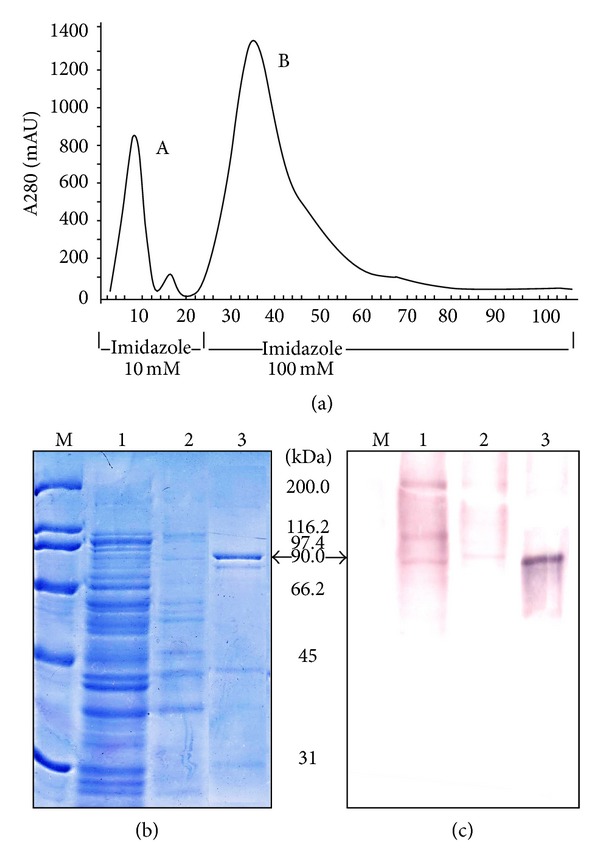
Purification and Western blot analysis of VacA. (a)  Purification of* H. pylori* VacA toxin by Ni^2+^affinity chromatography. The column was washed with 0.1 M Tris-HCl, pH 8.0, 0.3 M NaCl, 10 mM imidazole (*peak a*). VacA toxin was eluted with elution buffer (0.1 M Tris-HCl, pH 8.0, 0.3 M NaCl, 100 mM imidazole) (*peak b*). (b) SDS-PAGE (10% gel) analysis of* H. pylori* VacA toxin after IMAC purification. Lane M: broad range protein marker; lane 1: flow-through; lane 2: 10 mM imidazole wash; lane 3: 100 mM imidazole elution fraction of the protein. (c) Corresponding Western blot of* H. pylori* VacA toxin after IMAC purification. Western blot profile of the gel as seen in (a) using anti-VacA antibody on 10% SDS-PAGE. Lane M: broad range protein marker; lane 1: flow-through; lane 2: 10 mM imidazole wash; lane 3: 100 mM imidazole elution fraction of the protein.

The recombinant VacA protein was purified to near-homogeneity in a single step procedure by metal chelate affinity chromatography (IMAC). At a concentration of 10 mM imidazole, most host proteins were eluted from the affinity matrix and recombinant VacA protein was eluted from the column with 100 mM imidazole as a single peak (Figure 2(a)). Elution fractions analyzed on SDS-PAGE revealed a major protein band at 90 kDa and another minor protein band at ~88 kDa (Figure 2(b)). Subsequent Western blotting with anti-VacA rabbit polyclonal antiserum produced a single protein band at ~90 kDa (Figure 2(c)). The purity of VacA in the elution fraction was estimated to be >95%; however, the yield of the recombinant VacA protein was relatively low (<1 mg L^−1^ of bacterial culture).

### 3.3. VacA Apoptotic Activity

Apoptosis plays a major role in the pathogenic action of* H. pylori *[37]. Previous studies have established that a correlation exists between the development of duodenal ulcer in* H. pylori *infection and the level of apoptosis in antral mucosal epithelium [38]. Literature reports have also shown that the vacuolating toxin from* H. pylori *can increase the epithelial permeability of T84 and MDCK monolayers independent of its vacuolating activity and that* in vitro* infection of T84 intestinal epithelial cells with* H. pylori* can result in apoptosis [39–41]. Moreover, earlier studies have shown that exposure to VacA induces the degradation of tight junctions in MDCK cells [39]. The T84 and MDCK cell lines thus appear to represent interesting models to study the interaction of* H. pylori *with epithelial monolayers leading to apoptotic cell death.

**Figure 3:**
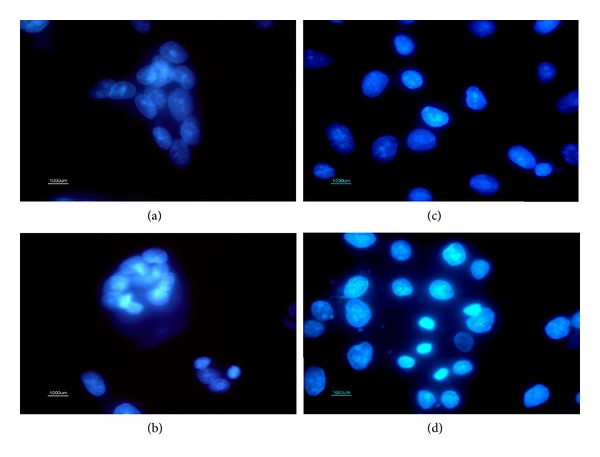
DAPI staining of intestinal epithelial cells (T84) and Madin-Darby Canine Kidney (MDCK) cells. (a) Control of untreated T84 cells showing normal nuclei. (b) T84 cells treated with VacA (150 µg mL^−1^) cells showing chromatin condensation and DNA fragmentation. (c) Control of untreated MDCK cells. (d) MDCK cells treated with VacA (150 µg mL^−1^) showing chromatin condensation and DNA fragmentation. DAPI was used at 1000-fold dilution in buffer and the magnification of images is 1000×.

Herein, apoptotic activity of the purified recombinant VacA toxin was detected by incubation of T84 and MDCK cells for 24 h in the presence of 150 µg mL^−1^ VacA protein and subsequent nuclear staining with DAPI analyzed by fluorescence microscopy. The DAPI-nuclear staining revealed an increase in the number of nuclei which showed cytopathic symptoms typical of apoptosis such as chromatin condensation as well as DNA fragmentation (Figure 3).

**Figure 4:**
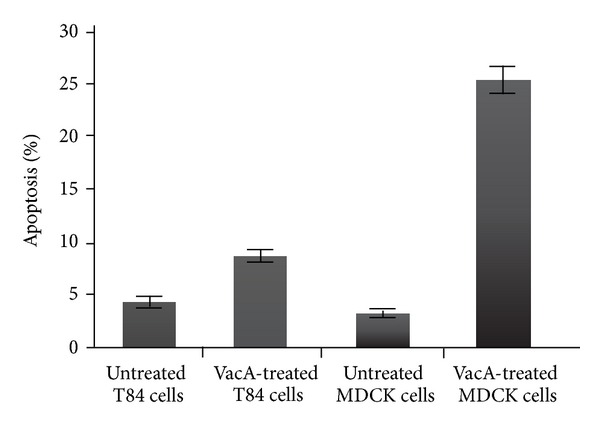
Percentage of apoptotic cells as observed after DAPI staining in T84 and MDCK cells. A mean of six pictures from each sample with at least 200 cells was counted for apoptosis-positive cells and the mean percentage of apoptotic cells was compared with the untreated control. Error bars represent SEM.

Incubation of T84 cells with VacA resulted in an increase in apoptosis of T84 cells with 8.2 ± 1.6% in VacA treated cells (mean ± SEM) compared with 4.2 ± 1.4% in the control cells with a significance *P* < 0.05 (Figure 4). VacA induced a marked apoptosis in MDCK cells with an increase of 25.4 ± 3.1% when compared to 3.6 ± 1.1% in control cells with *P* < 0.001 significance. Similar to T84 cells, presence of VacA induced apoptotic symptoms in treated MDCK cells even at a higher degree. Thus, MDCK cells appear to be significantly more sensitive to VacA than T84 cells.

An earlier study using HeLa cells suggested the existence of a nonapoptotic mechanism for Vac-induced cell death [42]. It is important to note that the experimental approach used herein does not allow conclusive differentiating between the induction of apoptosis or a programmed necrosis mechanism caused by VacA as proposed in a study using the gastric cell line AZ-521 [43]. While numerous reports have accumulated evidence that VacA-induced cell death involves the activation of caspases and the proapoptotic proteins Bax and Bak as well as the release of cytochrome C [44], Radin et al. suggested the existence of a necrotic pathway based on an observed release of LDH and the proinflammatory protein HMGB1 in VacA-treated AGS cells. It is noteworthy, however, that we have not observed an apparent rupture of the plasma membrane as typically seen with necrotic cells. It remains to be investigated in detail whether the cell-type specific release of proinflammatory proteins by gastric epithelial cells is caused by pyronecrosis or an apoptotic mechanism of cell death.

Differentiated T84 monolayers display high transepithelial resistance (TER) [45], a well-organized brush border, and the capacity to release IL-8 at the basal cell surface under adhesion with* H. pylori *[46]. Previous studies demonstrated that stimulation of T84 monolayers with* H. pylori *soluble extracts has dramatic effects on epithelial physiological balance and integrity [47]. Apical, but not basolateral, exposure of confluent monolayers of T84 cells to* H. pylori* extracts induces a rapid decrease in TER as well as the formation of domes. Domes are fluid-filled blister-like areas which form due to separation of the monolayer from the substrate, while the cells remain attached to each other. It was previously proposed that during an infection with* H. pylori *physiological gastric secretion in the antrum is impaired, eventually leading to the subsequent development of duodenal ulcer [47]. Although these findings suggest a correlation between the development of duodenal ulcer and the level of apoptosis in the antral mucosal epithelium, the precise molecular mechanism of the events leading to an accelerated disease development remains to be investigated.

## 4. Conclusion

We have successfully established the construction of a prokaryotic expression system for* vacA* gene from a clinical isolate and purification of the biologically active toxin. This is the first reported analysis of a VacA toxin from a patient sample obtained in Thailand. The isolate represents an s1/m2 allelic type and purified VacA toxin was able to induce apoptosis in two types of epithelial cell lines. This study could serve as an entry point to future investigations on genetic diversity within the* vacA* gene and their role for the differential pathogenicity of different strains of* H. pylori*.
